# Androgenic agricultural pollution suppresses immune function and alters reproductive allocation in eastern mosquitofish

**DOI:** 10.1093/conphys/coag052

**Published:** 2026-07-23

**Authors:** Upama Aich, Gabriel Melhado, Raiko Rafeeq, Morelia Camacho-Cervantes, Jack Manera, Bob B M Wong

**Affiliations:** Centre for Evolutionary Biology, School of Biological Sciences (M092), University of Western Australia, 35 Stirling Hwy, Crawley, WA 6009, Australia; School of Biological Sciences, Monash University, 25 Rainforest Walk, Clayton, VIC 3800, Australia; School of Biological Sciences, Monash University, 25 Rainforest Walk, Clayton, VIC 3800, Australia; School of Biological Sciences, Monash University, 25 Rainforest Walk, Clayton, VIC 3800, Australia; Instituto de Ciencias del Mar y Limnología, Universidad Nacional Autonoma de Mexico, Ciudad Universitaria, Mexico City 04510, Mexico; School of Biological Sciences, Monash University, 25 Rainforest Walk, Clayton, VIC 3800, Australia; School of Biological Sciences, Monash University, 25 Rainforest Walk, Clayton, VIC 3800, Australia

**Keywords:** Anabolic steroid, aquatic ecotoxicology, ecophysiology, evolution, Immunocompetence Handicap Hypothesis, immunosuppression, life-history trade-offs

## Abstract

Anthropogenic pollution poses a serious threat to wildlife. Particularly concerning are endocrine-disrupting chemicals (EDCs) due to their ability to mimic or interfere with hormone signalling. Given the fundamental role of hormones in regulating immunity and reproduction, androgenic EDCs are predicted to impact immunocompetence and associated life-history trade-offs. Yet, despite their potency and environmental ubiquity, the effects of androgenic EDCs on immune function and reproductive allocation remain poorly understood. Here, we tested how exposure to the androgenic agricultural pollutant 17β-trenbolone affects immunity and reproductive investment in eastern mosquitofish (*Gambusia holbrooki*). Adult mosquitofish were exposed to environmentally relevant concentrations of trenbolone (control: 0 ng/l, low: 11 ± 2.9 ng/l, high: 101 ± 13.6 ng/l) for 45 days. Immune function was assessed using phytohaemagglutinin (PHA)-induced skin swelling, white blood cell (WBC) counts and neutrophil–lymphocyte ratio (NLR), while male reproductive investment was quantified by sperm production. Trenbolone exposure significantly impaired immunity, reducing PHA-induced swelling by ~25%, decreasing total WBC counts and increasing NLRs, a biomarker of inflammation and immune response. In contrast, trenbolone exposure increased sperm number, and we found an overall negative correlation between immune response and sperm production. Our findings highlight how androgenic pollutants can simultaneously compromise immunity and alter reproductive allocation, enforcing evolutionary trade-offs that could negatively affect individual fitness and population resilience in contaminated environments.

## Introduction

Anthropogenic pollution poses a significant global threat to the health of humans and wildlife (Bernhardt *et al.*, 2017; Chen *et al.*, 2024). Especially concerning are endocrine-disrupting chemicals (EDCs), which can mimic, block or alter the synthesis, transport and metabolism of endogenous hormones (Diamanti-Kandarakis *et al.*, 2009; Zoeller *et al.*, 2012; Gore *et al.*, 2015). These pollutants, originating from industrial, agricultural and urban activities, have been linked to adverse effects on wildlife, including impairments in behaviour, development, reproduction and immune function—with potentially dire consequences for the viability and resilience of affected populations (Diamanti-Kandarakis *et al.*, 2009; Zoeller *et al.*, 2012; Martin *et al.*, 2025).

Broadly, EDCs with androgenic properties have historically received less research attention compared to their estrogenic counterparts, despite their similarly widespread presence and substantial role in regulating key physiological processes in vertebrates (Davey and Grossmann, 2016; Lagesson *et al.*, 2019). This knowledge gap is notable, as endogenous androgens, such as testosterone, are essential for male reproduction, including in spermatogenesis, the development and maintenance of secondary sexual traits and in mediating behaviour (O’Donnell *et al.*, 2015). Androgens are also potent modulators of the immune system, often exerting immunosuppressive effects that may influence the balance of resource allocation between immunity and reproduction (Folstad and Karter, 1992; Foo *et al.*, 2017). This androgen-mediated suppression of immune function forms the basis of the Immunocompetence Handicap Hypothesis, which posits that elevated androgen levels promote the expression of sexual traits while simultaneously reducing immune competence, thus maintaining the reliability of sexual signals to potential mates (Hamilton and Zuk, 1982; Folstad and Karter, 1992). Under this framework, individuals in good condition should be able to sustain high androgen levels and express costly sexual traits despite the associated immune costs (Foo *et al.*, 2017). Importantly, exposure to androgenic pollutants may externally elevate androgenic signalling and thereby alter, exaggerate or decouple this natural immune–reproductive trade-off.

Empirical support for androgen-mediated immunosuppression has been found across diverse vertebrate taxa. For instance, high androgen levels have been associated with increased parasite loads in three-spined sticklebacks (*Gasterosteus aculeatus*; Kurtz *et al.*, 2007), and reduced cell-mediated immune responses in zebra finches (*Taeniopygia guttata;*  Alonso-Alvarez *et al.*, 2007). Such findings highlight how androgen exposure can suppress investment in somatic maintenance, likely due to increased allocation in reproductive traits (Folstad and Karter, 1992; Foo *et al.*, 2017). However, both the magnitude and direction of androgen-immunity trade-offs can be species- and context-dependent, with some studies reporting partial or absent immunosuppression (reviewed in Roberts *et al.*, 2004; Foo *et al.*, 2017). This variability underscores the need for more experimental validation across taxa, and the Immunocompetence Handicap Hypothesis provides a central evolutionary framework for investigating how endogenous and environmental androgens, including those of anthropogenic origin, might disrupt fundamental life-history trade-offs.

In comparison to endogenous androgens, the ecological and physiological impacts of androgenic pollutants remain an emerging field, particularly their role in modulating the vertebrate immune system (Ankley *et al.*, 2018; Franke *et al.*, 2024). The few studies that have tested the effects of such androgens on animal immunity have reported complex and often multifaceted outcomes. For instance, synthetic androgens may impair cell-mediated immune responses by reducing antigen-specific immune responses, as evidenced by reduced delayed-type hypersensitivity responses in mice treated with the agricultural pollutant 17β-trenbolone (Hotchkiss and Nelson, 2007). However, in rainbow trout (*Oncorhynchus mykiss*), trenbolone did not affect fish cellular innate immunity but affected their humoral immune system (Massart *et al.*, 2015). These context-dependent results, likely stemming from the diverse receptor affinities and metabolic fates of different synthetic androgens (Li *et al.*, 2024), highlight the need for targeted empirical investigation in species often exposed to androgenic pollutants, particularly at ecologically relevant concentrations. Moreover, while immunity-reproduction trade-offs are theoretically predicted to strengthen under elevated androgen exposure (Hamilton and Zuk, 1982; Stearns, 1989; Davies *et al.*, 2024), few empirical studies have rigorously tested this prediction under field-realistic conditions. Understanding whether synthetic androgens impose life-history trade-offs is crucial for assessing their long-term ecological and evolutionary impacts.

Accordingly, we investigate how environmentally relevant exposure to a synthetic androgen, 17β-trenbolone, impacts immunity and reproductive investment in the eastern mosquitofish (*Gambusia holbrooki*). Mosquitofish are a widely distributed species found in temperate and subtropical regions worldwide (Koya and Iwase, 2004). Within this range, the species often inhabits environments contaminated by various pollutants, making it an increasingly important and ecologically relevant model organism in aquatic ecotoxicology (Toft *et al.*, 2004; Saaristo *et al.*, 2013). The anabolic steroid 17β-trenbolone, widely used in livestock as a growth promotant to enhance meat yields, is highly potent, environmentally persistent and frequently detected in aquatic habitats, including in surface waters and agricultural runoff. Environmental concentrations of trenbolone typically range from around 0.0013 to 20 ng/l in stream water and 0.0015 to 270 ng/l in feedlot run-off and lagoon water (Ankley *et al.*, 2003, 2018). While the reproductive effects of trenbolone have been studied in various aquatic vertebrates—such as, altered sex ratios in zebrafish (*Danio rerio*) and Japanese medaka (*Oryzias latipes*; 50 ng/l in Örn *et al.*, 2006), disruption of relationship between pre- and post-copulatory reproductive traits in eastern mosquitofish (11 ng/l in Tan *et al.*, 2021), and impacts on gonad development in frogs (2.7 and 27 μg/l in Rozenblut-Kościsty *et al.*, 2019)—its impacts on immune function remain largely unexplored (but see Massart *et al.*, 2015). Moreover, given the high affinity of trenbolone for the androgen receptor in fish (Ankley *et al.*, 2003; Lynch *et al.*, 2017), it is plausible that environmental exposure to this compound could impose the immunosuppressive effects associated with endogenous androgens and disrupt the balance between sexual investment and immunity.

We hypothesized that exposure to 17β-trenbolone would lead to immunosuppression in mosquitofish. Such immunosuppression may result in a reallocation of resources towards reproduction at the expense of immune function, thereby enforcing the trade-off predicted by the Immunocompetence Handicap Hypothesis. To test this prediction, adult eastern mosquitofish were chronically exposed to two different environmentally relevant concentrations of trenbolone alongside a control group for 45 days. Fish immune function was assessed through a suite of assays, including phytohaemagglutinin (PHA)-induced skin swelling, white blood cell (WBC) counts, and neutrophil–lymphocyte ratios (NLRs). As mosquitofish males rarely court and often mate by coercion, male reproductive investment was evaluated by measuring sperm number. By examining the interplay between exposure to a potent androgenic EDC and these key physiological parameters in a wild fish species, our study aims to provide novel insights into the ecological risks of agricultural pollutants and their potential to disrupt fundamental life-history trade-offs in aquatic animals.

## Materials and methods

### Animal collection and maintenance of stock fish

Eastern mosquitofish used in this study were collected from a freshwater lake located at Monash University's Clayton campus i(37°54′28″ S, 145°08′16″ E). Water sampling at this site has indicated no contamination with trenbolone (Envirolab Services, unpublished data). Following collection, fish were transported to the laboratory, where they were housed in large tanks (90 × 45 × 45 cm) containing carbon-filtered freshwater and were aerated by commercial air pumps (Resun LP100). The tanks also contained a 3 cm layer of gravel (7 mm grain size), rocks and aquatic plants (*Taxiphyllum barbieri*) to provide areas of shelter and enrichment. Water quality was maintained through weekly (20%) water changes. Fish were fed ad libitum with *Artemia* and commercial fish food (Aquasonic Nutra Xtreme C1).

### Exposure regime of experimental fish

After 2 weeks of acclimation, fish were randomly assigned to one of the three exposure treatments: control (no trenbolone), low trenbolone (measured concentration: 11 ± 2.9 ng/l) and high trenbolone (101 ± 13.6 ng/l). Each treatment group included three replicate populations (*n* = 32 fish per replicate, with a 1:1 sex ratio), totalling 288 fish across all treatments. The low- and high-trenbolone treatments reflect concentrations repeatedly detected in freshwater habitats, with the former representing common surface water concentrations of around 0.0013–20 ng/l in trenbolone-contaminated systems and the latter representing levels of 0.0015–270 ng/l typically found in heavily effluent-dominated waterbodies (Ankley *et al.*, 2018). The chosen exposure duration of 45 days was selected to allow for the detection of potential physiological effects of trenbolone on immunity and spermatogenesis in mosquitofish (Koya and Iwase, 2004; O’Donnell *et al.*, 2015). This period included 28 days of group housing exposure, followed by 17 days of individual housing to account for variation in sperm production in a social setting (O'dea *et al.*, 2014). For individual housing, fish were maintained in 2 l jars (12 × 23 cm, diameter × height), which enabled us to keep track of individual animal identity. Each jar had openings to allow air exchange. Each individual housing also contained gravel substrate (2 cm layer) and live vegetation (Java moss) transferred from their corresponding group-housing exposure tank to maintain environmental continuity, provide shelter and enrichment and minimize housing-related stress. These conditions were standardized across treatments. Water temperature (24 ± 1.0°C) was monitored daily, and fish were kept on a 12:12-h light:dark cycle during the housing and experimental procedures.

## Exposure monitoring and water analysis

For trenbolone exposure, the low and high treatment tanks received initial doses of trenbolone dissolved in 1 ml of ethanol (HPLC-grade, ≥99.99%) to reach the average measured concentrations. As 17β-trenbolone is lipophilic and requires an organic solvent for preparation, we used an ethanol solvent control rather than an untreated water-only control. All control tanks therefore received an initial dose of 1 ml ethanol and subsequent ethanol re-doses at the same time as the trenbolone exposure tanks. This design is consistent with standard ecotoxicological practice, as it allowed us to distinguish treatment effects from any potential solvent-related effects (Massart *et al.*, 2015; Rozenblut-Kościsty *et al.*, 2019; Orford *et al.*, 2024). Weekly ~20% water changes were performed with aged reverse osmosis water across all treatments to maintain water quality. Immediately after each water change, low and high trenbolone tanks were re-dosed with respective trenbolone stock solutions while the control tanks were re-dosed with ethanol throughout the exposure period to maintain our desired trenbolone concentrations and to keep tank handling standardized across treatments. The dosing solution was distributed across the tank and gently mixed with the exposure water to minimize disturbance to the fish and to ensure even dispersal. Water samples for analytical verification were collected ~24 h after dosing, allowing time for the trenbolone solution to disperse through the water. For individual housing, jars were filled with 2 l of treatment water and received 20% water changes weekly using treatment water to maintain respective trenbolone concentrations. Treatment concentrations were verified using high-performance liquid chromatography–tandem mass spectrometry (HPLC-MS–MS). Water samples (100 ml) were collected from exposure tanks, and analysed by a commercial environmental testing company (Envirolab Services, MPL Laboratories). To measure the water concentration, an eight-point calibration curve was prepared using 17β-trenbolone standards in unexposed aquarium water and analysed alongside experimental samples with 17α-trenbolone as a surrogate standard. Two transitions were monitored for 17β-trenbolone, with 271.2 → 199.3 used as the quantification ion and 271.2 → 165 used as the confirmatory ion (*sensu*  Orford *et al.*, 2024). The limit of quantification was 2 ng/l. Measured exposure concentrations are reported in Table 1.

**Table 1:** Measured 17β-trenbolone concentrations (ng/l) in exposure water during the experimental period

**Treatment**	**Sample size**	**Mean**	**SE**
Control	9	<2	0
Low	18	11.032	2.91
High	17	101.08	13.63

### Ethics approval

Experimental procedures were approved by the University’s Biological Sciences Animal Ethics Committee (approval number 35154, fish collection permit number NP1129).

### Morphological measures

Following the exposure period, mosquitofish were anaesthetized in an ice-cold water bath before being photographed and weighed. Individuals were photographed using a Nikon D5100 camera with a 60 mm lens, positioned at a 90° angle to the right lateral plane, with a reference scale and standardized lighting and camera conditions. We used ImageJ software to measure standard fish body length (snout to caudal peduncle). Fish body mass was recorded using a digital analytical scale (Scientech ZSA 210 scale; ±0.0001 g). As the fish were wild collected, their exact ages were unknown. However, all individuals used in the experiment were sexually mature young adults during the time of exposure. The females had an average standard body length (±SE) of 20.57 ± 0.19 mm and body mass of 0.146 ± 0.004 g (*n* = 159), while males had an average standard body length of 20.29 ± 0.17 mm and body mass of 0.134 ± 0.003 g (*n* = 117).

### Immune assays

i) PHA-mediated immune response

Immediately after photography, we used a PHA injection assay to measure the cell-mediated immune response of the mosquitofish, following previously published protocols (Iglesias-Carrasco *et al.*, 2019; Aich *et al.*, 2022). The PHA-stimulated challenge provides an effective evaluation of inflammation and cellular immune function and has previously been applied and validated in *G. holbrooki* (Iglesias-Carrasco *et al.*, 2019). First, we measured fish body thickness at the posterior end of the dorsal fin with a pressure-sensitive spessimeter (Mitutoyo 547-301; accuracy: 0.01 mm). Five consecutive measurements were taken prior to PHA injection, and the mean of these five measurements was recorded as the pre-injection thickness. We then injected a fixed volume of 0.01 mg PHA dissolved in 0.01 ml phosphate buffer solution into the left side of the fish at the point where body thickness had been measured. Fish were returned to their individual jars, and after 24 h, fish body thickness was re-measured five consecutive times, and the mean was recorded as the post-injection thickness. The difference between the pre- and post-injection values (i.e. swelling) defined the individual’s immune response to a PHA challenge (Iglesias-Carrasco *et al.*, 2019).

ii) Blood count

We counted WBCs in fish as an additional cell-mediated response to trenbolone exposure and immune challenge as haematological profiles are widely used to assess immune status and physiological condition across vertebrates, including fish (Paksuz *et al.*, 2009; Witeska *et al.*, 2022). Briefly, following the PHA-swelling measure, male fish were stripped of sperm (see sperm collection methods below), and all fish were humanely euthanized using MS-222. Blood samples were collected from fish hearts using venipuncture (by sterile injection needle), and blood smears were prepared immediately and air-dried. Subsequently, WBCs were stained by submerging the blood smears in Wright’s/Giemsa stain solution for 20 min before being washed with distilled water and allowed to dry. From each stained slide, we counted the total WBC in 10 field views of each blood smear at ×1000 magnification in a microscope. The number of WBCs counted per field of view was an average of 86.31 cells, with a minimum of 33, and a maximum of 204. WBC counts were done to test the immune capacity of fish in response to an immune challenge after exposure to different levels of trenbolone treatment. In addition, a separate independent count of the first 100 WBCs on each blood smear was performed to estimate the number of neutrophils and lymphocytes to calculate the NLR. The NLR acts as a biomarker to reflect the balance between two aspects of the immune system: acute and chronic inflammation (as indicated by the neutrophil count) and adaptive immunity (lymphocyte count) (Buonacera *et al.*, 2022).

### Sperm number

Following PHA-mediated immune measurement, sperm from male mosquitofish were extracted following established protocols (Aich *et al.*, 2022). To release the ejaculate of a male, a metal probe was used to swing the gonopodium forward, and gentle pressure was applied to the abdomen to induce sperm release. The stripped ejaculate was then transferred to a 1.5 ml Eppendorf tube containing 100–1300 μl of activation solution (150 mM KCl and 2 mg/ml bovine serum albumin), with the quantity of solution used depending on the amount of ejaculate released by each male. This solution was then gently resuspended and vortexed for 1 min to break up the spermatozeugmata. Finally, 20 μl of 10% formalin and 10 μl of trypan blue were added to the solution for cell fixation and staining.

The estimated sperm count was performed using an improved Neubauer chamber under a microscope at 40× magnification (Motic B1-220E-SP). For this, a 10 μl aliquot of sperm sample was loaded into each well of the haemocytometer and allowed to settle for 5 min. Then, the sperm number was counted for 10 of the 0.2 × 0.2 mm squares, five per haemocytometer chamber. The total sperm number was estimated by the mean of these 10 counts multiplied by their dilution factor in millilitre and divided by 0.000004 ml (the volume of the 0.2 × 0.2 mm squares).

### Statistical analysis

First, to test the effect of trenbolone on the immune response of mosquitofish, we used multiple linear mixed-effects models to analyse the effect of trenbolone on three response variables: PHA response, total WBC count and NLR. For each response variable, the model included trenbolone treatment, fish sex and their two-way interaction as fixed factors. Body size was added as a covariate, and the trenbolone treatment tank was added as a random factor to account for any potential tank effect. If the addition of tank as a random factor did not explain any variation in the models and resulted in singularity issues, we ran linear models for traits by dropping the random factor to aid in model fit, and found that the results remained unchanged (see Supplementary Tables S1 and S2). Fish WBC count was log-transformed to meet the assumptions of our models. To test the effects of trenbolone on blood count (total WBC and NLR), we also added individual PHA response as a covariate in the model, as PHA challenge can independently affect WBC counts (Hawley *et al.*, 2023).

Second, to test the effect of trenbolone on the relationship between immunity and male sperm number, we fitted a linear mixed-effects model that included trenbolone treatment, PHA-induced swelling response and their two-way interaction as fixed factors. Sperm number was log-transformed to meet the assumptions of our models. As only a subset of males produced sperm, we fitted a generalized linear mixed-effects model with binomial family to test whether treatment affected the likelihood of sperm production. In both cases, fish body size was added as a covariate, and the treatment tank was added as a random factor.

In all cases, if the two-way interaction was non-significant, it was removed from the model to test the main effects of fixed factors on our response variables. *Post hoc* pairwise comparisons were run using the emmeans package (Lenth and Lenth, 2018) to identify differences between treatment levels (Table S3).

All models were run by setting alpha = 0.05 and performing two-tailed tests using the R package lme4 (Bates *et al.*, 2014) in R v. 4.4.3 (R Core Team, 2025). We checked the distribution of residuals using the DHARMa package (Hartig and Hartig, 2017) and plot function to ensure they met the model assumption. Model predictors were tested for significance using the Anova function in the car package (Fox and Weisberg, 2018).

## Results

### Analytical verification of 17β-trenbolone concentration

To account for left-censoring of 17β-trenbolone due to the method detection limit (MDL = 2 ng/l), all trenbolone samples that fell below the MDL (17β-trenbolone treatment *n* = 12) were included in the analysis as the MDL divided by 2, following Antweiler (2015). The mean measured concentrations (±SE) for the low and high 17β-trenbolone treatments during the exposure period were 11.03 ± 2.91 ng/l and 101.08 ± 13.63 ng/l, respectively (Table 1 and Fig. S1, Supplementary data), with temporal variation in measures likely due to the known fate of 17β-trenbolone in aquatic systems, including degradation, photolysis, sorption to solids or organic material and biological uptake (Ankley *et al.*, 2018). No 17β-trenbolone was detected in the control treatment tanks.

### PHA response

**Figure 1:**
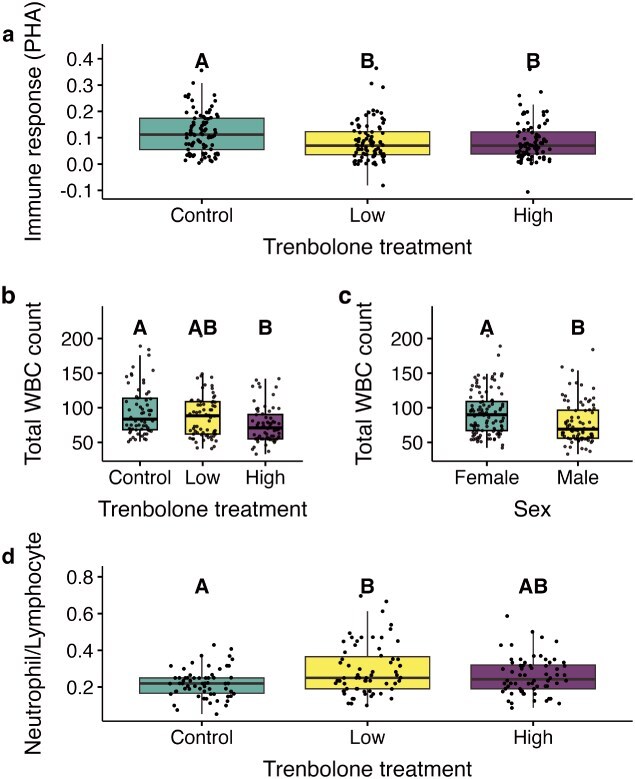
The effect of trenbolone exposure and sex on immune function in mosquitofish. (a) Immune response to a PHA challenge across trenbolone treatments (*n* = 89 control, 88 low and 81 high). (b) Total WBC count across trenbolone treatments (*n* = 62 control, 66 low and 64 high). (c) Total WBC count by sex (*n* = 109 females and 83 males). (d) NLR across trenbolone treatments (*n* = 62 control, 66 low and 64 high). Boxplots show the median (horizontal line), interquartile range (box) and data range (whiskers). Individual data points are jittered. Letters above plots indicate significant differences (*P* < 0.05) between groups from Tukey’s *post hoc* tests.

There was no significant interaction between trenbolone treatment and sex on mosquitofish PHA response (*F*_2_,_251_ = 0.241, *P* = 0.786; Table 2a). However, trenbolone exposure had a direct effect on the PHA challenge (*F*_2_,_253_ = 5.288, *P* = 0.006). Specifically, fish from both low and high trenbolone treatment had significantly lower PHA responses than the control group (Fig. 1a and Table 3a). We found no significant effect of sex (*F*_1_,_253_ = 0.938, *P* = 0.334) or body length (*F*_1_, _253_ = 1.23, *P* = 0.268) on mosquitofish PHA response (Table 3a).

**Table 2:** Parameter estimates of linear models predicting immune response to (a) PHA challenge, (b) WBC count and (c) NLR in mosquitofish

**Predictor**	**Estimate**	**SE**	***t*-statistic**	***P***	***F*-statistic**	***P* (ANOVA)**
**(a) PHA response**						
(Intercept)	0.07	0.048	1.459	0.146	2.128	0.146
TreatmentH	−0.037	0.016	−2.389	**0.018**		
TreatmentL	−0.032	0.014	−2.214	**0.028**	3.659	**0.027**
SexMale	−0.013	0.016	−0.789	0.431	0.623	0.431
Body_length_mm	0.003	0.002	1.164	0.245	1.356	0.245
TreatmentH:SexMale	0.013	0.023	0.567	0.572		
TreatmentL:SexMale	−0.002	0.023	−0.08	0.937	0.241	0.786
**(b) WBC count**						
Intercept	4.395	0.257	17.093	**<0.0001**	292.177	**<0.001**
TreatmentH	−0.137	0.085	−1.617	0.108		
TreatmentL	0.013	0.080	0.163	0.870	1.914	0.150
SexMale	−0.032	0.091	−0.351	0.726	0.123	0.726
PHA _response	0.363	0.388	0.937	0.350	0.877	0.350
Body_length_mm	0.004	0.012	0.300	0.765	0.090	0.765
TreatmentH:SexMale	−0.142	0.123	−1.155	0.250		
TreatmentL:SexMale	−0.203	0.125	−1.625	0.106	1.388	0.252
**(c) NLR**						
Intercept	0.156	0.084	1.859	*0.065*	3.456	*0.065*
TreatmentH	0.038	0.028	1.361	0.175		
TreatmentL	0.079	0.026	3.033	**0.003**	4.611	**0.011**
SexMale	0.032	0.030	1.089	0.277	1.186	0.277
PHA _response	−0.051	0.126	−0.401	0.689	0.161	0.689
Body_length_mm	0.003	0.004	0.703	0.483	0.494	0.483
TreatmentH:SexMale	−0.003	0.040	−0.078	0.938		
TreatmentL:SexMale	−0.011	0.041	−0.265	0.791	0.038	0.963

The model outputs are shown for interactive fixed effects of trenbolone treatment (control, low and high, abbreviated) and sex (male and female), with treatment: control and sex: female being the reference categories. Positive estimates indicate a greater immune response. The standard error (SE), *t*-values and *P*-values were obtained from the model summary. *F*-statistic values were calculated with the analysis of variance (ANOVA) test. Significant results are marked in bold.

**Table 3:** Parameter estimates of linear models predicting immune response to (a) PHA challenge, (b) WBC count and (c) NLR in mosquitofish

**Predictor**	**Estimate**	**SE**	***t*-statistic**	***P***	***F*-statistic**	**P (ANOVA)**
**(a) PHA response**						
(Intercept)	0.072	0.046	1.552	0.122		
TreatmentH	−0.031	0.011	−2.695	**0.008**		
TreatmentL	−0.032	0.011	−2.881	**0.004**	5.288	**0.006**
SexMale	−0.009	0.009	−0.968	0.334	0.938	0.334
Body_length_mm	0.002	0.002	1.109	0.268	1.23	0.268
**(b) WBC count**						
Intercept	4.485	0.251	17.859	**<0.0001**		
TreatmentH	−0.197	0.062	−3.179	**0.002**		
TreatmentL	−0.069	0.062	−1.108	0.269	5.264	**0.006**
SexMale	−0.152	0.051	−2.982	**0.003**	8.891	**0.003**
PHA_response	0.3	0.386	0.777	0.438	0.603	0.438
Body_length_mm	0.002	0.012	0.159	0.874	0.025	0.874
**(c) NLR**						
Intercept	0.161	0.081	1.98	**0.049**		
TreatmentH	0.037	0.02	1.818	*0.071*		
TreatmentL	0.075	0.02	3.708	**0.0002**	6.886	**0.001**
SexMale	0.027	0.016	1.657	*0.099*	2.745	*0.099*
PHA _response	−0.053	0.125	−0.426	0.670	0.182	0.670
Body_length_mm	0.003	0.004	0.678	0.499	0.459	0.499

The model outputs are shown for main effects of trenbolone treatment (control, low and high, abbreviated) and sex (male and female), with treatment: control and sex: female being the reference categories. Positive estimates indicate a greater immune response. The standard error (SE), *t*-values and *P*-values were obtained from the model summary. *F*-statistic values were calculated with the ANOVA test. Significant results are marked in bold. Italicised *P*-values indicate weak statistical trends (0.05 < P < 0.10)

### WBC count

We found no interactive effect of trenbolone treatment and sex on the total WBC count (*F*_2_,_183_ = 1.388, *P* = 0.252; Table 2b). However, trenbolone exposure had a direct effect on total WBC count (*F*_2_,_185_ = 5.264, *P* = 0.006). Compared to the control group, fish from high-trenbolone exposure had reduced WBC counts. However, neither control and low, or low and high-trenbolone fish differed in their WBC counts (Fig. 1b and Table 3b). We also found a direct effect of sex on total WBC count (*F*_1_,_185_ = 8.891, *P* = 0.003). Specifically, female mosquitofish had more WBCs than males, irrespective of their exposure treatment (Fig. 1c and Table 3b). Finally, neither PHA response (*F*_1_,_185_ = 0.603, *P* = 0.438) nor body length (*F*_1_,_185_ = 0.025, *P* = 0.874) had any detectable effect on fish WBC count.

### Neutrophil–lymphocyte ratio

There was no detectable interactive effect of trenbolone treatment and sex on the NLR (*F*_2_,_183_ = 0.038, *P* = 0.963; Table 2c). However, trenbolone exposure had a dose-specific effect on the NLR (*F*_2_,_185_ = 6.886, *P* = 0.001). Specifically, fish from the low-trenbolone exposure group had a higher NLR than the control fish, but the high-trenbolone group did not differ significantly from the control or low-trenbolone fish (Fig. 1d and Table 3c). Sex did not have any significant effect on the NLR (*F*_1_,_185_ = 2.745, *P* = 0.099). Likewise, neither the PHA challenge (*F*_1_,_185_ = 0.182, *P* = 0.670) nor body length (*F*_1_,_185_ = 0.459, *P* = 0.499) had any detectable effect on the NLR.

### Immunocompetence-reproduction trade-off

**Figure 2:**
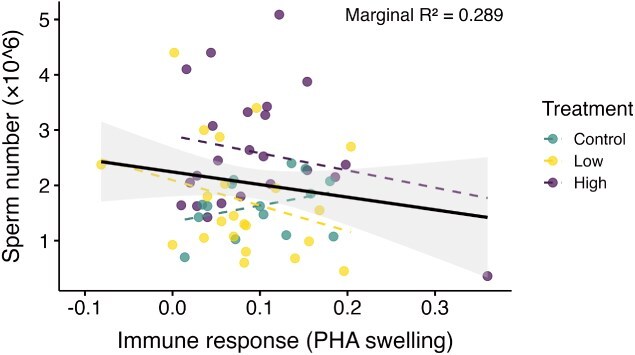
Relationship between sperm number and immune response (PHA swelling) in male mosquitofish. The solid line shows the overall effect of immune response across all treatments, and treatment-specific dashed lines represent within-treatment trends for visualization. Points represent individual males from each treatment (*n* = 17 control, 22 low and 24 high); shaded area shows 95% CI.

We found no significant interaction between immune response and trenbolone treatment on sperm number in male mosquitofish (*χ*^2^_2_ = 3.609, *P* = 0.165; Table S4). However, both immune response and trenbolone exposure had direct effects on sperm number (Table 4). In particular, we found a negative association between immune response and sperm number (*χ*^2^₁ = 5.853, *P* = 0.016), indicating a trade-off consistent with the Immunocompetence Handicap Hypothesis (Fig. 2 and Table 4). Trenbolone exposure was also found to significantly increase overall sperm number (*χ*^2^₂ = 10.182, *P* = 0.006; Fig. S2). *Post hoc* comparisons revealed a weak trend for high-trenbolone exposure group to have higher sperm numbers than the low-trenbolone group (estimate = 0.50 ± 0.16 SE, *P* = 0.061); by contrast, there was no difference in sperm number between either the control versus low, or control versus high trenbolone fish (Table S5). Exposure treatment also did not affect the probability of male sperm production (*χ*^2^₂ = 2.12, *P* = 0.345). Lastly, larger males had a higher chance of producing sperm (*χ*^2^₁ =8.763, *P* = 0.003), and they also produced significantly more sperm (*χ*^2^₁ = 6.316, *P* = 0.012).

**Table 4:** Parameter estimates of models testing the direct effects of immune response, and trenbolone treatment on sperm number in male mosquitofish

**Predictor**	**Estimate**	**SE**	***t*-statistic**	***P***	***χ*** ^**2**^	***P* (ANOVA)**
(Intercept)	11.939	1.022	11.683	**<0.00001**		
TreatmentH	0.337	0.174	1.939	*0.091*		
TreatmentL	−0.159	0.176	−0.902	0.394	10.182	**0.006**
Immune_response	−2.198	0.909	−2.419	**0.019**	5.853	**0.016**
Body_length_mm	0.121	0.048	2.513	**0.015**	6.316	**0.012**

The model outputs are shown for main effects of trenbolone treatment (control, low and high, abbreviated) and immune response, with treatment: control being the reference category. Positive estimates indicate a greater sperm count. Standard errors (SE), *t*-values and *P*-values are from model summaries, while Chi-square values are from ANOVA tests. Significant results are in bold. Italicised *P*-values indicate weak statistical trends (0.05 < P < 0.10)

## Discussion

EDCs are increasingly recognized as major drivers of physiological disruption in aquatic fauna, yet how androgenic pollutants mediate fundamental trade-offs between immunity and reproductive investment in wild fish remains largely unknown. Our study provides compelling evidence that exposure to environmentally relevant concentrations of the widespread androgenic endocrine-disrupting pollutant 17β-trenbolone compromises multiple facets of immune function in the eastern mosquitofish, while simultaneously enhancing male reproductive investment. We demonstrate that androgenic pollutant exposure may enforce the fundamental evolutionary trade-off between immunity and reproduction, as predicted by the Immunocompetence Handicap Hypothesis. In so doing, this study is the first to empirically test whether exposure to a synthetic androgenic endocrine disruptor can induce evolutionary trade-offs between reproduction and immunity in a wild fish species at environmentally relevant concentrations, with potential consequences for population health in contaminated aquatic environments.

### Decrease in PHA-swelling response

Our observed reduction in PHA-induced skin swelling in mosquitofish exposed to trenbolone aligns with a substantial body of literature demonstrating the immunosuppressive effects of androgens and androgenic EDCs in fish and other vertebrates (Davey and Grossmann, 2016). While fewer studies have specifically examined the effect of trenbolone on cell-mediated immunity in fish, similar immunosuppressive effects on PHA responses have been reported in European starlings (*Sturnus vulgaris*; Duffy *et al.*, 2000) and in Mediterranean lacertid lizards (*Psammodromus algirus* and *Acanthodactylus erythrurus*; Belliure *et al.*, 2004) when treated with testosterone. Moreover, studies on rainbow trout (*O. mykiss*) exposed to trenbolone have shown alterations in humoral immunity, including inhibited lysozyme activity and modulation of cytokine expression (Massart *et al.*, 2015). The suppression of innate inflammatory responses and subsequent alteration of adaptive immune capacity are expected to impair the clearance of bacterial pathogens (Hornef *et al.*, 2002; Nedeva, 2021), which could lead to increased parasite loads and decreased survival in wild fish populations. Such immunosuppression in the presence of androgenic pollution would likely reduce individual survival and overall population viability (Austin and Austin, 2016), potentially exacerbated by other environmental stressors, such as elevated temperatures and hypoxia (Lagesson *et al.*, 2019; Franke *et al.*, 2024). Future studies are needed to directly test for synergistic effects of multiple environmental challenges to better understand the full extent of immunosuppression in rapidly changing anthropogenic environments (see Lagesson *et al.*, 2019; Orford *et al.*, 2024).

### Reduction in total WBC count

We found a significant decrease in total WBC count in mosquitofish exposed to high levels of trenbolone, which further supports the immunosuppressive effects of androgenic compounds in aquatic vertebrates. This pattern is consistent with existing literature demonstrating androgen-induced immunosuppression in various fish species, including suppressed T-cell proliferation and reduced cytokine responses in rainbow trout (Massart *et al.*, 2015) and seabream (*Sparus aurata*; Sánchez-Hernández *et al.*, 2013). Interestingly, we observed consistently higher WBC counts in female mosquitofish compared to males, independent of trenbolone treatment and body size. This sex-specific difference in immune investment is potentially driven by differential hormonal profiles and life-history strategies (Zuk and Stoehr, 2010; Foo *et al.*, 2017). Indeed, existing literature across a range of taxa suggests that females typically invest more heavily in immune function due to differing reproductive costs and trade-offs compared to males (reviewed in Klein and Flanagan, 2016; Kelly *et al.*, 2018). This mechanism of sex-specific response is further supported by the notion that sex chromosomes and gonadal hormones modulate the number and functions of immune cells, resulting in differences in immune response (Hoffmann *et al.*, 2023; Dunn *et al.*, 2024). The combined evidence of lowered WBC counts and reduced PHA-induced swelling in trenbolone-exposed mosquitofish indicates a diminished capacity to mount effective immune responses, which may increase pathogen susceptibility and exacerbate population-level health risks, particularly in males inhabiting contaminated environments. Nevertheless, we acknowledge that these assays do not capture all dimensions of immune competence. Therefore, future studies combining whole-organism assays with cellular, molecular and pathogen-challenge approaches could provide a more complete mechanistic understanding of how androgenic pollutants alter immune function in fish.

### Dose-dependent effect on NLR

We also found a dose-specific effect of trenbolone exposure on the NLR, with low concentrations significantly increasing the NLR in mosquitofish, indicative of elevated physiological stress. The NLR is a widely recognized biomarker of stress and immune disruption across taxa, with an increased ratio generally reflecting inflammation and an imbalance between innate and adaptive immunity (Buonacera *et al.*, 2022). The increased neutrophil numbers at low trenbolone concentrations may indicate an activated innate immune response. Yet, the suppression of PHA-induced swelling in the trenbolone-exposed individuals in our study suggests a disruption in the hormonal signalling required for immune cell migration to the site of challenge. On the contrary, lymphocyte suppression in our trenbolone-exposed fish further points to compromised adaptive immunity, which could impair immunological memory and reduce the host’s ability to respond to subsequent pathogen exposures (Gubbels Bupp and Jorgensen, 2018). Similar androgenic effects have been observed in other species, with testosterone treatment leading to elevated neutrophils in rats (Scalerandi *et al.*, 2018), and high androgen levels resulting in reduced lymphocyte counts in male Azorean rock-pool blennies (*Parablennius parvicornis*; Ros *et al.*, 2006). Likewise, a higher NLR observed in Brazilian sandperch (*Pinguipes brasilianus*; Sueiro *et al.*, 2020) exposed to anthropogenic pollutants parallels our findings in mosquitofish. The lack of a further increase in NLR at high trenbolone concentrations suggests a potential non-linear endocrine response (Boettcher *et al.*, 2011), a common pattern reported in endocrine toxicology, where high doses can trigger compensatory feedback mechanisms or receptor desensitization (Calabrese and Baldwin, 2001; Vandenberg *et al.*, 2012). Given the scarcity of studies examining synthetic androgen effects on fish NLR (Grayfer *et al.*, 2018; Buonacera *et al.*, 2022), further research is needed to understand the mechanistic pathways through which trenbolone alters stress physiology in aquatic vertebrates.

### Immunocompetence-reproductive trade-offs

We found that mosquitofish from trenbolone exposure showed patterns of elevated sperm production, along with significant reductions in immune parameters, providing empirical support for a trade-off between investment in sexually selected traits and immune function, mediated by sex hormones under the Immunocompetence Handicap Hypothesis (Hamilton and Zuk, 1982; Folstad and Karter, 1992; Foo *et al.*, 2017). Critically, the negative association between PHA-induced swelling and sperm number indicates that trenbolone, acting as an exogenous androgen (Ankley *et al.*, 2018), may enforce this fundamental physiological trade-off. Similar androgen-mediated costs have been demonstrated across taxa, including increased parasite loads in brightly coloured passerines (Hamilton and Zuk, 1982), reduced immunity in testosterone-treated eastern fence lizards (*Sceloporus undulatus*; Cox *et al.*, 2005) and suppressed innate immune responses in androgen-treated sticklebacks (Kurtz *et al.*, 2007). Elevated androgen levels in wild meerkats also enhance reproductive success while simultaneously increasing parasitism and immunosuppression (Davies *et al.*, 2024).

By applying the Immunocompetence Handicap Hypothesis in the context of testing the impacts of wildlife exposure to anthropogenic pollution, our study highlights a critical but underappreciated ecological risk: androgenic EDCs may override natural physiological allocation of resources, forcing organisms to prioritize reproduction at the expense of immune defence (Roberts *et al.*, 2004; Brannelly *et al.*, 2025). Such disruption could intensify mortality from infections or disease, particularly in contaminated environments (Kurtz *et al.*, 2007; Austin and Austin, 2016), and drive selection for reduced sensitivity to androgenic EDCs or alternative immune-reproductive allocation strategies (Ros *et al.*, 2006; Foo *et al.*, 2017; Chen *et al.*, 2024). Additionally, pollutant-induced immunosuppression may exacerbate vulnerability to other environmental stressors, including rising temperatures and emerging pathogens, further compromising population resilience. These evolutionary and ecological consequences underscore the urgent need for research into how chronic androgen exposure reshapes natural selection and host–parasite dynamics in ecosystems increasingly burdened by anthropogenic pollution (Cerini *et al.*, 2023). Future research should investigate whether chronic androgen exposure leads to heritable shifts in immune or reproductive allocation, how pollutant-induced trade-offs interact with co-occurring environmental stressors, and whether phenotypic plasticity in hormone sensitivity can buffer organisms against such pressures. If EDCs like trenbolone act as significant selective agents shaping life-history strategies in polluted environments, understanding these complex interactions is crucial for effective ecological risk assessment and the development of robust strategies to mitigate the harmful effects of chemical pollution in aquatic ecosystems.

From a conservation and environmental management perspective, our findings highlight the importance of considering immune function as a sublethal endpoint in assessments of endocrine-disrupting pollutants. Current contaminant risk frameworks for endocrine-active substances often focus on population-relevant apical endpoints, such as survival, mortality and reproduction (Marty *et al.*, 2017; Ankley *et al.*, 2018), yet immunological endpoints remain less commonly incorporated despite their ecological relevance (Casanova-Nakayama *et al.*, 2011; Kataoka and Kashiwada, 2021). Immune suppression may reduce disease resistance and population resilience before population-level declines become detectable (Austin and Austin, 2016; Franke *et al.*, 2024). Incorporating immune measures into freshwater monitoring could therefore improve ecological risk assessment for androgenic pollutants, particularly in agricultural catchments, where contaminants may interact with warming, hypoxia and pathogen exposure.

## Supplementary Material

Web_Material_coag052

## Data Availability

Data and code for the MS can be accessed via Figshare https://doi.org/10.6084/m9.figshare.29554136.
