# Supplementary material for: Androgenic agricultural pollution suppresses immune function and alters reproductive allocation in eastern mosquitofish
Source: Conserv Physiol. 2026 Jul 23;14(1):coag052. doi: 10.1093/conphys/coag052 (PMC13397028; doi:10.1093/conphys/coag052)
Supplement: Web_Material_coag052 [file web_material_coag052.zip › TrenImmune_SuppMaterial_revised V2 clean.pdf]

Androgenic agricultural pollution suppresses immune function and alters reproductive allocation in eastern mosquitofish

Supplementary material

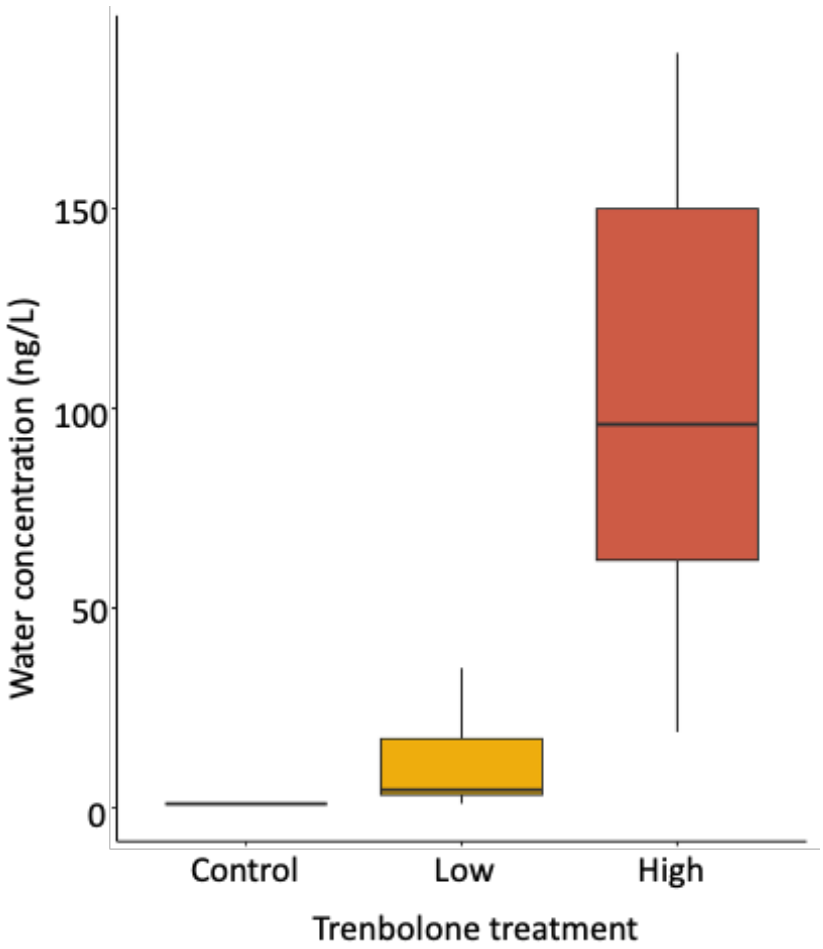

Fig S1. Boxplot shows the median (horizontal line) and interquartile range of trenbolone concentration recorded during the exposure period for the three exposure treatments (*n* = 9 control, 18 low, and 17 high).

Table S1. Parameter estimates of linear mixed effect models predicting the effect of trenbolone treatment on a) PHA response, b) white blood cell (WBC) count, and c) neutrophil-lymphocyte ratio in mosquitofish. The model output is shown **for interactive effects** of trenbolone treatment (control, low, and high, abbreviated), and sex (male, female) with the covariate body size. Positive estimates indicate a greater immune response. Standard errors (SE) and P-values were obtained from the model summary, while chi-square values and their P values were calculated with the likelihood ratio test. Significant results are marked in bold.

Predictor	Estimate	SE	t-value	*P*	χ^2	*P (χ2)*
a) PHA response						
TreatmentH	-0.037	0.016	-2.389	**0.018**		
TreatmentL	-0.032	0.014	-2.214	**0.028**	7.318	**0.026**
SexMale	-0.013	0.016	-0.789	0.431	0.623	0.430
Body_length_mm	0.003	0.002	1.164	0.245	1.356	0.244
TreatmentH:SexMale	0.013	0.023	0.567	0.572		
TreatmentL:SexMale	-0.002	0.023	-0.08	0.937	0.482	0.786
b) WBC count						
TreatmentH	-0.137	0.085	-1.617	0.108		
TreatmentL	0.013	0.08	0.163	0.870	3.328	0.148
SexMale	-0.032	0.091	-0.351	0.726	0.123	0.726
PHA _response	0.363	0.388	0.937	0.35	0.877	0.349
Body_length_mm	0.004	0.012	0.300	0.765	0.09	0.764
TreatmentH:SexMale	-0.142	0.123	-1.155	0.250		
TreatmentL:SexMale	-0.203	0.125	-1.625	0.106	2.776	0.250
c) Neutrophil-lymphocyte ratio						
TreatmentH	0.038	0.028	1.361	0.175		
TreatmentL	0.079	0.026	3.033	**0.003**	9.223	**0.011**
SexMale	0.032	0.030	1.089	0.277	1.186	0.277
PHA _response	-0.051	0.126	-0.401	0.689	0.161	0.689
Body_length_mm	0.003	0.004	0.703	0.483	0.494	0.483
TreatmentH:SexMale	-0.003	0.040	-0.078	0.938		
TreatmentL:SexMale	-0.011	0.041	-0.265	0.791	0.075	0.963

Table S2. Parameter estimates of linear mixed effect models predicting immune response to a) PHA response, b) white blood cell (WBC) count, and c) neutrophil-lymphocyte ratio in mosquitofish. The model outputs are shown for **main effects** of trenbolone treatment (control, low, and high; abbreviated) and Sex (male and female), with Treatment: control and Sex: female being the reference categories. Positive estimates indicate a greater immune response. The standard error (SE), t-values and P-values were obtained from the model summary. Chi-square values were calculated with the likelihood ratio test. Significant results are marked in bold.

**Predictor**	**Estimate**	**SE**	**t-value**	***P***	**χ^2**	***P (χ2)***
a) PHA response						
TreatmentH	-0.031	0.011	-2.695	**0.008**		
TreatmentL	-0.032	0.011	-2.881	**0.004**	10.575	**0.005**
SexMale	-0.009	0.009	-0.968	0.334	0.938	0.333
Body_length_mm	0.002	0.002	1.109	0.268	1.230	0.267
b) WBC count						
TreatmentH	-0.197	0.062	-3.179	**0.002**		
TreatmentL	-0.069	0.062	-1.108	0.269	10.528	**0.005**
SexMale	-0.152	0.051	-2.982	**0.003**	8.891	**0.003**
PHA_response	0.3	0.386	0.777	0.438	0.603	0.437
Body_length_mm	0.002	0.012	0.159	0.874	0.025	0.874
c) Neutrophil-Lymphocyte ratio						
TreatmentH	0.037	0.02	1.818	*0.071*		
TreatmentL	0.075	0.02	3.708	**<0.001**	13.772	**0.001**
SexMale	0.027	0.016	1.657	*0.099*	2.745	*0.098*
PHA_response	-0.053	0.125	-0.426	0.670	0.182	0.670
Body_length_mm	0.003	0.004	0.678	0.499	0.459	0.498

Table S3. Parameter estimates and test statistics for pairwise comparisons of trenbolone exposure on the immune response to a) PHA challenge, b) white blood cell (WBC) count, and c) neutrophil-lymphocyte ratio in mosquitofish.

**Contrast**	**Estimate**	**SE**	**Df**	**T-Ratio**	***P***
a) PHA response					
Control - High	0.031	0.011	253	2.695	**0.020**
Control - Low	0.032	0.011	253	2.881	**0.012**
High - Low	0.001	0.012	253	0.120	0.992
b) WBC count					
Control - High	0.197	0.062	185	3.179	**0.005**
Control - Low	0.069	0.062	185	1.108	0.511
High - Low	-0.129	0.061	185	-2.118	0.089
c) Neutrophil-Lymphocyte ratio					
Control - High	-0.037	0.02	185	-1.818	0.166
Control - Low	-0.075	0.02	185	-3.708	**0.001**
High - Low	-0.038	0.02	185	-1.936	0.132

Table S4. Parameter estimates of linear mixed models testing relationships between immune response and sperm number in male mosquitofish under trenbolone exposure. Estimates are shown for **interactive effects** between trenbolone treatment (control, low, high; abbreviated) and immune response, with control as the reference category. Positive estimates indicate a greater immune response. Standard errors (SE), t-values, and P-values are from model summaries, while F-statistics and Chi-square values are from Anova tests. Significant results are in bold.

**Predictor**	**Estimate**	**SE**	**t-statistic**	***P***	**χ^2**	***P (Anova)***
(Intercept)	11.669	1.019	11.452	**<0.001**	131.15	**<0.001**
TreatmentH	0.812	0.299	2.715	**0.009**		
TreatmentL	0.263	0.302	0.872	0.389	9.796	**0.007**
Immune_response	1.628	2.236	0.728	0.470	0.530	0.466
Body_length_mm	0.116	0.048	2.419	**0.019**	5.852	**0.016**
TreatmentH: Immune_response	-4.793	2.571	-1.864	0.068		
TreatmentL: Immune_response	-4.329	2.726	-1.588	0.118	3.609	0.165

Table S5. Parameter estimates and test statistics for pairwise comparisons of trenbolone exposure on the sperm number in mosquitofish.

**Contrast**	**Estimate**	**SE**	**Df**	**T-Ratio**	***P***
Control - High	-0.337	0.174	6.87	-1.932	0.201
Control - Low	0.159	0.177	7.11	0.898	0.658
High - Low	0.496	0.159	4.76	3.120	*0.061*

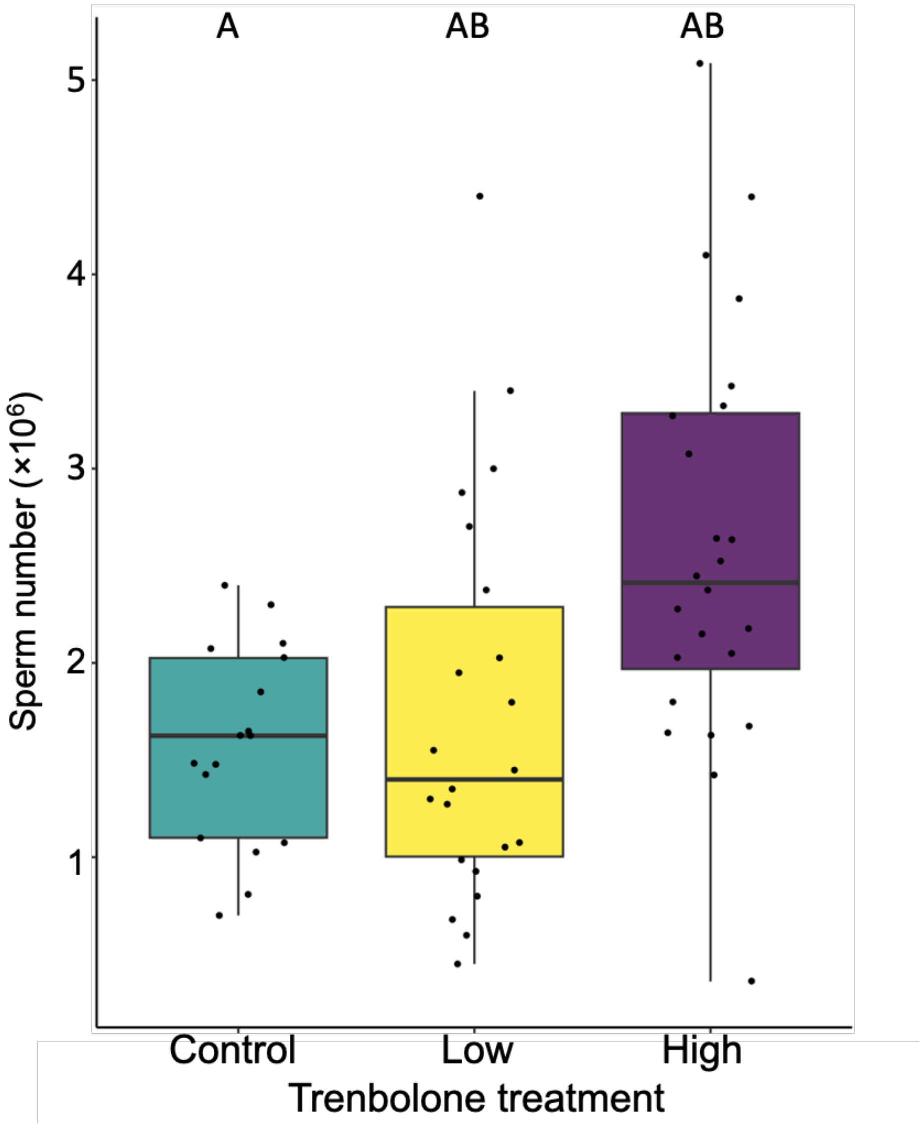

Fig S2. Effect of trenbolone treatment (*n* =17 control, 22 low, and 24 high) on fish sperm number. Boxplots show the median (horizontal line) and interquartile range of raw data points. Letters indicate significant differences using Tukey's tests.
